# Pretransplant Donor-Specific Anti-HLA Antibodies and the Risk for Rejection-Related Graft Failure of Kidney Allografts

**DOI:** 10.1155/2020/5694670

**Published:** 2020-01-29

**Authors:** Michiel G. H. Betjes, Kasia S. Sablik, Henny G. Otten, Dave L. Roelen, Frans H. Claas, Annelies de Weerd

**Affiliations:** ^1^Department of Nephrology & Transplantation, Erasmus Medical Center, Rotterdam, Netherlands; ^2^Laboratory of Translational Immunology, University Medical Center Utrecht, Utrecht, Netherlands; ^3^Department of Immunohematology and Blood Transfusion, Leiden University Medical Center, Leiden, Netherlands

## Abstract

**Background:**

The presence of donor-specific antibodies (DSAs) against HLA before kidney transplantation has been variably associated with decreased long-term graft survival. Data on the relation of pretransplant DSA with rejection and cause of graft failure in recipients of donor kidneys are scarce.

**Methods:**

Patients transplanted between 1995 and 2005 were included and followed until 2016. Donor-specific antibodies before transplantation were determined retrospectively. For cause, renal transplant biopsies were reviewed.

**Results:**

Pretransplant DSAs were found in 160 cases on a total of 734 transplantations (21.8%). In 80.5% of graft failures, a diagnostic renal biopsy was performed. The presence of pretransplant DSA (DSApos) increased the risk of graft failure within the first 3 months after transplantation (5.2% vs. 9.4%) because of rejection with intragraft thrombosis (*p* < 0.01). One year after transplantation, DSApos recipients had an increased hazard for antibody-mediated rejection at 10 years (9% DSAneg vs. 15% DSApos, *p* < 0.01). One year after transplantation, DSApos recipients had an increased hazard for antibody-mediated rejection at 10 years (9% DSAneg vs. 15% DSApos, *p* < 0.01). One year after transplantation, DSApos recipients had an increased hazard for antibody-mediated rejection at 10 years (9% DSAneg vs. 15% DSApos,

**Conclusions:**

Pretransplant DSAs are a risk factor for early graft loss and increase the incidence for humoral rejection and graft loss but do not affect the risk for T cell-mediated rejection.

## 1. Introduction

The presence of donor-specific antibodies (DSAs) against HLA molecules is a risk factor for humoral rejection after kidney transplantation. The introduction of the complement-dependent cytotoxicity (CDC) test has been a major step forward in excluding high-risk donor-acceptor combinations [1]. The CDC recognizes the most harmful DSA, which are able to bind complement and subsequently lyse donor cells carrying HLA on their cell surface.

In recent years, a number of other assays has become available which are able to recognize antibodies against HLA with a much greater level of sensitivity [2]. The flow cytometry assay using beads coated with a single HLA molecule is the most sensitive and now widely used to identify DSA [2–4]. However, the clinical importance is still a matter of debate as conflicting results are published on the impact on long-term graft survival [5–8]. In addition, some studies show a significant impact on graft survival within the first months after transplantation while other studies do not [9, 10].

The presence of pretransplant DSA identifies patients which are sensitized to allogeneic HLA, e.g., by pregnancy, blood transfusions, or a previous transplantation. Therefore, pretransplant DSA may identify patients with both an increased risk for antibody-mediated rejection as well as T cell-mediated rejection [11]. To date, a number of publications have shown the increased risk for recipients with pretransplant DSA to develop an acute antibody-mediated rejection shortly after transplantation and subsequent decreased graft survival [9, 12, 13]. However, data on long-term biopsy proven rejection and cause of graft failure defined by renal biopsy, in relation to pretransplant DSA, are lacking.

Within the Dutch National Profiling Consortium of Antibody Repertoire and Effector functions (PROCARE), pretransplant DSAs were measured retrospectively in all recipients of a kidney transplant in the period 1995–2005 in the Netherlands [14, 15]. This offers a unique data set as clinical decision-making, and immune suppressive medications were not based on knowledge about pretransplant DSA status.

In this study, the pretransplant DSA data were combined with the clinical and renal histopathological data of our transplantation center and the impact of pretransplant DSA on the type of graft rejection and cause of graft loss was analyzed.

## 2. Materials and Methods

This study included all 734 kidney transplantations performed between January 1995 and December 2005 at the Erasmus Medical Center in the Netherlands. The last follow-up date was January 1, 2017.

The pretransplantation protocol used PRA assessment and CDC testing. A positive CDC crossmatch of the recipient with the potential donor, but not the percentage of PRA, was an absolute contraindication for transplantation. In every case of transplantation, the CDC crossmatch was negative with current and historic peak sera. PRA did not influence the medical policy regarding the type of induction therapy or subsequent immune suppressive medication used. All transplantations were ABO compatible.

Bead assay defined DSA was not taken into consideration in the matching procedure within the period of patient inclusion. Informed consent for data collection and use of leftover sera was obtained from all subjects. Sera used for DSA determination were assessed within the larger cohort of the PROCARE study comprising all Dutch kidney transplant centers [14]. Recipients of a donor kidney gave their informed consent to store their clinical data in the NOTR (The Dutch Organ Transplantation Registry). The use of clinical data and assessment of donor-specific antibodies in stored serum samples was approved by the Research Ethics Committee for Biobanks and the Medical Ethics Committee of the University Medical Center Utrecht.

The baseline and clinical follow-up transplantation data were retrieved from the Netherlands Organ Transplant Registry (NOTR), which was over 99% complete for our center at time of this study. Graft failure is defined as loss of kidney function when the patient returns to dialysis or receives a retransplant. For the analysis of rejection-free survival and rejection-related graft failure, the recipients who died with a functioning graft (141 patients) were censored at the time of death and patients lost to follow-up censored at the time of the visit to the outpatient clinic (28 patients, 3.8%). Primary nonfunction of the graft is defined as the persistent need for dialysis after transplantation or the absence of graft function in preemptively transplanted end-stage renal disease patients.

Time from transplantation to the first renal biopsy showing rejection was taken to calculate rejection-free survival.

The renal biopsies were performed because of progressive loss of graft function and scored in accordance with the Banff criteria 2015. Rejection episodes were classified as cellular (T cell-mediated rejection), humoral (antibody-mediated rejection), or mixed-type rejection. The latter type of rejection presented a small group (*n* = 10), and for statistical analysis, these cases were combined with the humoral rejections.

A cellular rejection was treated with prednisolone 1000 mg for 3 days, and in case of insufficient response, antithymocyte immunoglobulin was given. Humoral rejections were treated with prednisolone 1000 mg for 3 days with intravenous immunoglobulins (1 gr/kg). Plasmapheresis was rarely performed, and in case of rapid declining graft function without sufficient response to therapy, antithymocyte immunoglobulin was given.

## 3. Detection and Definition of Donor-Specific HLA Antibodies

The presence of HLA antibodies (HLA-Abs) in the pretransplant sera was assessed as described previously [16]. In brief, sera were tested for the presence of HLA class I and class II antibodies using Lifecodes LifeScreen Deluxe and Lifecodes SAB assay class-I and -II kits (Immucor Transplant Diagnostics, Stamford, CT). The LABScan 100 flow analyzer (One Lambda, Canoga Park, CA) was used for data acquisition. The cutoff level was defined according to manufacturer's instructions. The presence of SAB-DSA was assigned by comparing the SAB-HLA-A/B/DR/DQ antibody specificities on the serological level with the broad-level HLA typing of the donor. DSAs to HLA-C, HLA-DP, and DQA1 were not assessed as within the studied period of transplantation, and HLA typing of the kidney donors did not routinely include these HLA loci.

The number of DSAs per individual, the maximum MFI of DSA, and the cumulative MFI of DSA were calculated and used for the relation with transplant outcome.

## 4. Statistical Analysis

Differences in patient, donor, and transplant characteristics between the DSA-positive and -negative group were assessed by Fisher's exact test for categorical variables and Mann–Whitney *U* test for continuous variables. All *p* values were 2-tailed.

Death-censored graft loss and incidence of rejection were assessed by Kaplan–Meier analysis with log-rank statistics for difference between strata. All data were analyzed for the first 3 months, the first year after transplantation, and after the first year of transplantation, given the clear differences in clinical events early and late after transplantation.

Univariate Cox proportional hazards analysis was used to identify clinical and demographic variables associated with rejection and graft survival. Variables with a *p* value of <0.1 were considered for further analysis by stepwise forward regression to calculate hazard ratios and corresponding confidence intervals. pH assumption of variables was tested by visual inspection of log-minus log graphs and further tested by assessment of time-dependency using the Cox regression with time-dependent covariate module in SPSS. All variables met the demands of pH unless stated otherwise. Interaction terms that met statistical significance (*p* < 0.05) were included in the multivariate model. Normal probability plots were made, and presence of significant correlations was assessed. Absence of collinearity in the model covariates was formally assessed by calculating the variance inflation factor. Statistical analysis was performed with software IBM SPSS statistics 21.

## 5. Results

### 5.1. Baseline Characteristics

The clinical and transplant characteristics of recipients and kidney stratified according to the presence of pretransplant DSA (DSApos versus DSAneg recipients) are given in Table 1. Pretransplant DSAs were present in 160 out of 734 (21.8%) transplantations performed. The majority of DSApos recipients had either anti-HLA class I or II antibodies (77.5%). The group of DSApos patients had significantly more retransplantations (50%). DSApos recipients received relatively more frequently a deceased donor kidney compared to the DSAneg recipients (58.8% vs. 44.1%) and had a significantly different male/female ratio in both the recipients and kidney donors. The mean total number of HLA mismatches was similar for recipients of living or a deceased donor kidney. The average number of DSA's per individual was 1.3 ± 0.08, the maximum MFI of DSA 7320 ± 4612, and the cumulative MFI of DSA 8206 ± 5092. A triple immunosuppressive regimen consisting of steroids, cyclosporine or tacrolimus, and mycophenolate mofetil or azathioprine was given initially in the large majority of patients. Only 7.08% of patients received induction therapy, virtually all with an IL-2 receptor-blocking antibody. A total of 383 graft failures were recorded (50.2% of transplantations in the DSAneg group and 59.4% in the DSApos group, *p*=0.04). Death with functioning graft occurred in 141 patients, and in 195 cases out of the 242 death-censored graft failures (80.5%), a diagnostic renal biopsy was performed. Graft failure because of rejection was diagnosed in more than half of these cases, with a significantly higher frequency of ABMR-related graft failure in the DSApos group (11.9% vs. 5.4%, *p* < 0.001).

### 5.2. Early Acute Rejection and Graft Failure in Recipients with and without Pretransplant DSA

**Figure 1:**
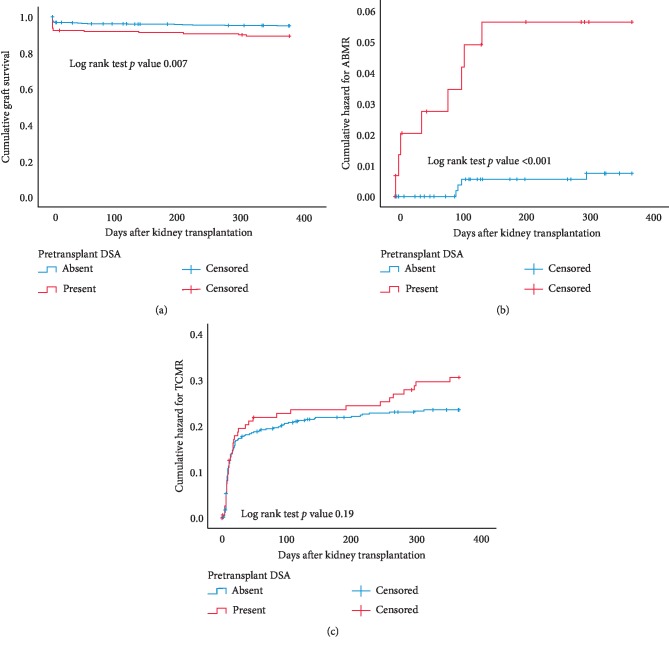
Kaplan–Meier analysis of graft survival censored for death within the first year after transplantation (a) of recipients with or without pretransplantation of donor-specific anti-HLA antibodies (DSA). (b, c) show the cumulative hazard of antibody-mediated rejection (ABMR) and T cell-mediated rejection (TCMR) for recipients within the first year after transplantation according to their status of pretransplant DSA (present or absent). *p* values are obtained by the log rank test statistics pairwise over strata.

Death-censored graft loss within the first year after transplantation, particular within the first months, was significantly higher in the recipients with pretransplant DSA (Figure 1(a)). Also, the cumulative hazard of ABMR was higher in the DSApos group (Figure 1(b)). More detailed analysis of early graft failure and acute rejection within the first 3 months after kidney transplantation showed an overall incidence of 6.1% of graft failure, 18.1% TCMR, and 0.7% ABMR (Table 2). Graft failure censored for death with a functioning graft at 3 months was higher in the DSApos group (8.1% vs. 3.8%, *p*=0.03). This was caused by a significant higher incidence of primary nonfunctioning grafts in which the majority could be attributed to acute rejection with severe arteritis. In 7 out of 10 cases from the DSAneg group, but none of the cases of the DSApos group, this was accompanied by tubulointerstitial rejection (*p*=0.02). Instead, renal biopsies in the DSApos group frequently showed intra-arterial thrombi or thrombotic microangiopathy (Table 2). Only in the DSApos group, 5 cases of intrarenal thrombosis were observed without the presence of arteritis or glomerulitis. These findings indicate that circulating pretransplant DSA are a risk factor for the development of early severe arteritis and intrarenal arterial thrombosis, leading to graft failure.

The cumulative hazard for TCMR was similar for the DSApos and DSAneg groups (Figure 1(c)).

Cox regression analysis showed that only the presence of pretransplant DSA was highly associated with ABMR within the first year after transplantation (Table 3). Pretransplant DSA, receiving a deceased donor kidney and donor age, significantly increased the risk for early graft failure (Table 3). The type of DSA (anti-HLA I, II, or in combination) was not significantly related to ABMR or early graft failure (data not shown).

### 5.3. Long-Term Risk for Rejection in relation to Pretransplant DSA

**Figure 2:**
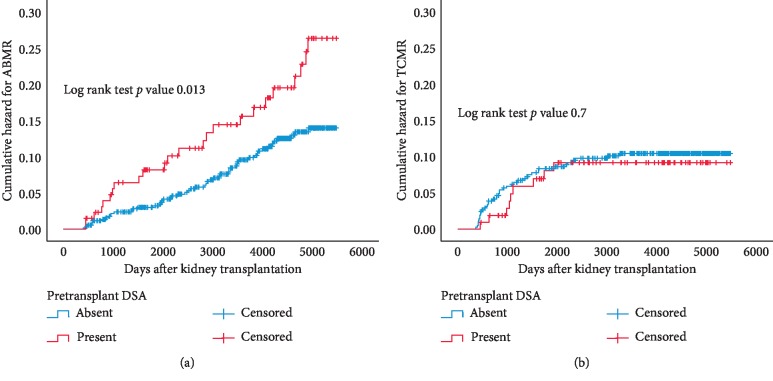
Kaplan–Meier analysis of the cumulative hazard for antibody-mediated rejection (ABMR) and T cell-mediated rejection (TCMR) after the first year after transplantation according to their status of pretransplantation donor-specific anti-HLA antibodies (DSA present or absent). *p* values are obtained by log rank test statistics pairwise over strata.

The presence of preexistent DSA significantly increased the incidence of ABMR after kidney transplantation (Figure 2(a)) with a cumulative hazard at 10 years of 9% in the DSAneg group vs. 15% in the DSApos group (*p*=0.01). The type of DSA (anti-HLA I, II, or in combination) was not related to the incidence of ABMR (data not shown). In addition, the average number of DSA's per individual, the maximum MFI of DSA, and the cumulative MFI of DSA were not related to late rejection.

Univariate and multivariate analyses showed that the presence of pretransplant DSA was significantly associated with ABMR, with a significant interaction between DSA presence and receiving a deceased donor kidney (Table 4).

In contrast, the long-term incidence of TCMR (patients more than 12 months after transplantation) became close to zero 5 years after transplantation. The cumulative hazard of TCMR in the patient groups with or without pretransplant DSA was overlapping (Figure 2(b)).

### 5.4. Long-Term Risk for Graft Failure in relation to Pretransplant DSA

**Figure 3:**
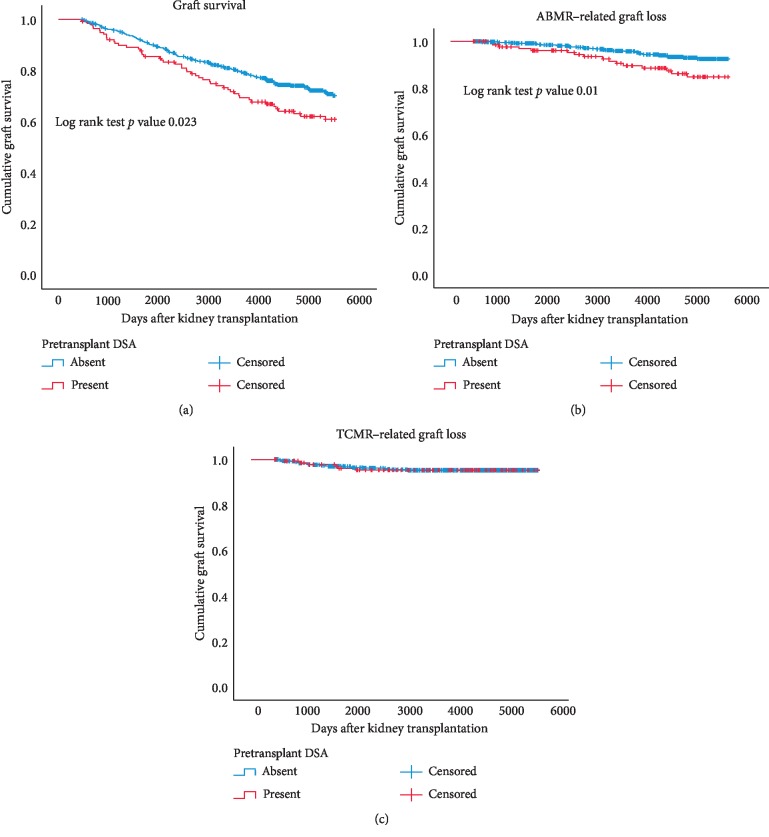
Kaplan–Meier analysis of graft survival censored for death after the first year after transplantation (a). Graft loss because of antibody-mediated rejection (ABMR) or T cell-mediated rejection (TCMR) is shown in b and c. Separate survival curves are made for recipients with or without pretransplantation donor-specific anti-HLA antibodies (DSA present or absent). *p* values are obtained by log rank test statistics pairwise over strata.

The group of patients with pretransplant DSA had a significantly decreased death-censored graft survival (Figure 3(a)). Graft survival at 10 years was 79% in the DSAneg recipients and 69% in the DSApos recipients (*p*=0.02). The graft loss because of ABMR was significantly increased in den DSApos recipients (*p*=0.01, Figure 3(b)). After five years of posttransplantation, ABMR was diagnosed as the cause for graft failure in 47% of renal biopsies performed.

Multivariable analysis showed that age of donor, recipient age, and the interaction term for pretransplant DSA and donor type were significantly associated with the long-term risk of graft failure (Table 5). The type of DSA (anti-HLA I, II, or in combination) was not significantly related to the incidence of ABMR-related graft loss (data not shown). In addition, the average number of DSA's per individual, the maximum MFI of DSA, and the cumulative MFI of DSA were not related to late rejection and or graft failure in accordance with previous publications [17, 18].

In contrast, graft failure because of TCMR was most frequent in the first years after transplantation but became increasingly rare thereafter and was not influenced by the presence of pretransplant DSA (Figure 3(c)).

## 6. Discussion

The results of this study show that pretransplant DSA significantly increase the incidence of ABMR and ABMR-related graft failure but not of TCMR.

The short-term consequences of pretransplant DSA after kidney transplantation are an increase in severe vasculitis with thrombosis leading to graft loss, in particular in recipients of a deceased donor kidney. Our data also indicate that pretransplant DSA can result in intrarenal thrombosis without vasculitis. However, we cannot exclude the possibility of the sampling error, and isolated vasculitis lesions may have been present. Nevertheless, pretransplant DSA are clearly associated with intragraft thrombosis early after transplantation, which obviously contributes to graft loss. Three months after transplantation, the combination of vasculitis with multiple thrombi was not observed anymore. Graft loss associated with pretransplant DSA was predominantly observed in recipients of a deceased donor kidney. This may be caused by increased immunogenicity of the deceased donor kidney expressing more HLA and adhesion molecules on endothelial and tubular cells in combination with an increased vulnerability for severe vascular damage [19–21]. In line with this concept, Haller et al. recently showed that pretransplant DSA are particularly detrimental in patients with delayed graft function [22].

Pretransplant DSAs were predominantly found in patients, which were sensitized by a previous transplantation and therefore more frequently found in recipients of a deceased donor kidney. It is to be expected that allosensitization of T cells is also increased in the DSApos patients resulting in patients more prone to TCMR after transplantation. However, the incidence of TCMR was not affected by the presence of pretransplant DSA in this study and was previously even reported to be decreased [12]. This may be explained by the fact that the direct T cell response to allogeneic HLA antigens is predominantly involved in TCMR and not the indirect alloreactive T cell response [23]. The latter pathway is involved in the regulation of a humoral anti-HLA immune response.

Some publications have reported on the outcome of routine kidney allograft biopsies in patients with preexistent DSA and described histopathological signs of antibody-mediated rejection, specifically microvascular inflammation, in over 50% of all kidney allograft biopsies at 12 months [12, 24]. In addition, the presence of ABMR lesions at 1 year after transplantation is a risk factor for allograft loss [25]. However, not all grafts are affected by circulating DSA and when affected have a variable degree of microvascular inflammation and progression to graft loss. This indicates that other factors like complement binding capacity of the DSA and activation of the clotting system leading to intrarenal thrombotic microangiopathy or activation of immune cells are important contributing factors [26].

After the first year of transplantation, pretransplant DSA significantly increased the risk for ABMR and was associated with decreased overall graft survival. The latter was largely due to an increase in ABMR-related graft failure with a significant interaction between pretransplant DSA and deceased donor kidney. This is in accordance with a previous publication of the PROCARE study which showed a stronger negative effect of pretransplant DSA on deceased donor graft survival [15]. The significant interaction between pretransplant DSA and a deceased donor kidney on the risk of ABMR and graft failure after the first year is of interest. The explanation can only by hypothetical but may indicate that the activated endothelium of the deceased kidney transplant has a boosting effect on the (humoral) immune response which has a detrimental effect on long-term outcome.

Of note, this specific interaction was not confirmed in a recent publication from Germany reporting about a large number of kidney transplantations with or without pretransplant DSA [27]. However, both studies indicate that pretransplant DSAs are potentially harmful to the graft and should be avoided if possible resulting in better graft survival of both deceased and living donor kidneys.

In this study, we were unable to identify a relation between the MFI value of the DSA and the risk for ABMR or graft loss. Other groups did find a significant relation between a high MFI value and the risk for ABMR in recipients receiving a deceased donor kidney [28, 29]. However, these studies included a significant percentage of patients with either a historical or current positive CDC, and all patients, including the patients considered to be in the low risk-low MFI group, were receiving T cell depletion with a DSA lowering strategy (plasmapheresis or immunoabsorption).

The relative contribution of TCMR to graft loss was almost absent beyond 5 years after transplantation. These findings are in line with several reports identifying ABMR as a major cause of late graft loss [30–32].

A limitation of our study is the lack of information on newly formed DSA after transplantation. Also, within the period of transplantation, few patients received induction therapy and the data are therefore difficult to extrapolate to the current practice. However, this also offers an advantage as our findings are not obscured or biased by the use of different regimes of induction therapy. Clearly, within the current era of transplantation, the potential deleterious effect of DSA is recognized, and a recipient preferably does not receive a kidney in the presence of DSA or receives an intensified induction therapy with at least T cell depletion. However, our data also indicate that the majority of patients with pretransplant DSA do not have an increased risk for ABMR-related graft loss.

Nevertheless, kidney transplantation in the presence of DSA should be avoided if possible. The increased risk for early graft loss, particularly in case of a deceased donor kidney, should be balanced against a possible substantial increase in waiting time.

Another limitation is that, during the study period, the clinical practice of taking a diagnostic renal biopsy, the definition of humoral rejection, and treatment schedules all have changed. These changes could not be adequately accounted for, and therefore, their effect on the results of our study is essentially unknown.

## 7. Conclusions

The presence of pretransplant DSA increases the risk for ABMR and graft loss but is not associated with a changed incidence of TCMR. These findings are important for risk stratification of patients awaiting a donor kidney and the use of desensitization protocols.

## Figures and Tables

**Table 1:** Clinical characteristics of recipients and kidney donors stratified for pretransplant donor-specific antibodies to HLA.

	DSAneg, *N* = 574	DSApos, *N* = 160	*p* value
Age recipient ± SEM	46.2 ± 0.6	43.8 ± 1.0	0.064
Age donor ± SEM	47.2 ± 0.6	45.7 ± 1.1	0.19
Donor male sex	67.2%	40.6%	<0.001
Recipient male sex	43.7%	57.5%	0.002
Deceased donor	44.1%	58.8%	0.001
Living donor: related/unrelated	36.9%/19.0%	30.6%/10.6%	0.09
Cold ischaemia time in hours	10.2 ± 0.4	13.8 ± 0.9	0.2
Retransplantation	9.1%	49.7%	<0.001
PRA historic ± SEM	11.2% ± 0.8	43.3% ± 2.8	<0.001
PRA current ± SEM	2.9% ± 0.4	26.2% ± 2.5	<0.001
Total HLA mismatches (median)	3	3	0.4
DSA HLA class I only	—	35%	
DSA HLA class II only	—	42.5%	
DSA HLA class I and II	—	22.5%	
Induction therapy yes/no	35/539	17/143	0.055
Anti-IL-2 receptor antibody	33	17	0.047
T cell depleting antibody	2	0	>0.99
Initial immune suppression			
Steroids	93.0%	86.3%	>0.99
Tacrolimus/ciclosporin	60.3%/38.6%	59.3%/37.5%	>0.99
MMF/azathioprine	69.7%/0.5%	75.6%/0.0%	>0.99
Sirolimus	8.4%	7.5%	>0.99
Others	4.5%	3.1%	>0.99
Follow-up time (median years)	11.6	11.2	0.13
Total number of graft loss	288	95	0.040
Death with functioning graft	117	24	0.14
Surgery-related graft loss	3	0	>0.99
Cases with diagnostic renal biopsy^1^	134 (78.4%)	61 (85.9%)	0.21

PRA: panel reactive antibodies; DSA: donor-specific antibodies. ^1^% of total number of graft losses excluding cases of death with functioning graft.

**Table 2:** Characteristics of acute rejection and graft failure within the first 3 months after transplantation for recipients stratified for donor-specific antibodies for HLA.

	No pretransplant DSA (*n* = 574)	Pretransplant DSA (*n* = 160)	*p*value
Number of TCMR	102	31	0.64
Number of ABMR	0	5	0.0005
Total number of graft losses	30 (5.2%)	15 (9.4%)	0.06
Primary nonfunctioning graft	18 (3.1%)	12 (7.5%)	0.02
Causes of graft loss			
Death with functioning graft	8 (1.4%)	2 (1.2%)	>0.99
Vasculitis	10 (1.7%)	5 (3.1%)	0.33
Vasculitis with thrombi in arterioles	1 (0.2%)	3 (1.9%)	0.03
Vasculitis with tubulointerstitial rejection	7 (1.2%)	0 (0.0%)	0.35
Thrombi/TMA without vasculitis	1/0 (0.2%)	4/1 (3.1%)	0.002
Renal vein/artery thrombosis	3/1 (0.7%)	1 (0.6%)	>0.99
Acute tubular necrosis	2 (0.5%)	1 (0.6%)	>0.99
Others	3 (0.5%)	1 (0.6%)	>0.99
Surgery-related complications	2 (0.3%)	0 (0.0%)	>0.99

DSA: donor-specific anti-HLA antibodies; TCMR: T cell-mediated rejection; ABMR: antibody-mediated rejection; TMA: thrombotic microangiopathy.

**Table 3:** Univariate and multivariate Cox regression analysis for graft loss and rejection in the first year after transplantation.

	*p* value	Hazard ratio	95% CI
Univariate analysis for graft loss first year (45 events)^1^			
Male sex recipient	0.98	1.01	0.56–1.81
Age recipient (per year)	0.57	0.99	0.97–1.02
Age donor (per year)	0.005	1.03	1.01–1.06
Deceased donor kidney	<0.001	7.32	3.10–17.3
Previous transplant	0.23	1.51	0.77–2.98
Number of HLA mismatches	0.14	1.15	0.95–1.39
PRA current	0.11	1.63	0.89–2.96
PRA peak serum	0.69	0.88	0.46–1.67
DSA present	0.009	2.22	1.22–4.06
DSA^*∗*^deceased donor kidney	0.56	1.96	0.21–18.45

Multivariate analysis for graft loss first year			
DSA present	0.042	1.88	1.02–3.44
Age donor	0.003	1.04	1.01–1.06
Deceased donor kidney	<0.001	6.91	2.91–16.39

Univariate analysis for ABMR within first year (12 events)			
Male sex recipient	0.72	1.24	0.39–3.89
Age recipient (per year)	0.63	1.01	0.97–1.05
Age donor (per year)	0.87	1.00	0.96–1.04
Deceased donor kidney	0.79	1.17	0.38–3.62
Previous transplant	0.49	1.58	0.43–5.84
Number of HLA mismatches	0.23	1.25	0.87–1.78
PRA current	0.48	1.01	0.99–1.03
PRA peak serum	0.37	1.01	0.99–1.03
DSA present	0.001	7.70	2.32–25.57
DSA^*∗*^deceased donor kidney	0.64	0.57	0.051–6.23

^1^Numbers of events are graft losses censored for death with functioning graft. Total graft loss within the first year is 70. ^*∗*^Interaction term for variables.

**Table 4:** Univariate and multivariate Cox regression analysis for ABMR after the first year of transplantation.

	*p* value	Hazard ratio	95% CI
Univariate analysis for ABMR after 1 year (*n* = 658, 77 events)			
Male sex recipient	0.82	1.01	0.56–1.81
Age recipient (per year)	0.056	0.98	0.97–1.00
Age donor (per year)	0.63	1.00	0.99–1.02
Deceased donor kidney	0.73	0.92	0.59–1.45
Previous transplant	0.14	1.48	0.87–2.51
Number of HLA mismatches	0.071	0.87	0.75–1.01
PRA current pos	0.081	1.51	0.95–2.41
PRA peak serum pos	0.96	1.02	0.60–1.70
Pretransplant DSA present	0.014	1.83	1.13–2.96
DSA^*∗*^deceased donor kidney	0.001	2.58	1.51–4.42

Multivariate analysis for ABMR after 1 year			
DSA^*∗*^ deceased donor kidney	0.006	4.36	1.51–12.55

^*∗*^Interaction term for variables.

**Table 5:** Univariate and multivariate Cox regression analysis for overall graft loss and ABMR-related graft loss after the first year of transplantation.

	Graft loss after first year (184 events)			ABMR-related graft loss (52 events)		
Univariate analysis for graft loss	*p* value	Hazard ratio	95% CI	*p* value	Hazard ratio	95% CI
Male sex recipient	0.99	1.00	0.75–1.34	0.81	1.06	0.62–1.84
Age recipient (per year)	0.009	0.98	0.98–0.99	0.052	0.98	0.91–1.00
Age donor (per year)	0.013	1.01	1.00–1.03	0.13	1.02	0.99–1.036
Deceased donor kidney	0.014	1.43	1.07–1.92	0.37	1.28	0.74–2.20
Previous transplant	0.010	1.56	1.11–2.19	0.024	1.99	1.09–3.63
Number of HLA mismatches	0.94	1.00	0.91–1.10	0.23	0.89	0.75–1.70
PRA current pos	0.096	1.30	0.95–1.77	0.14	1.52	0.86–2.67
PRA peak serum pos	0.54	0.90	0.65–1.25	0.50	1.25	0.64–2.44
DSA present	0.024	1.45	1.05–2.01	0.011	2.09	1.18–3.71
DSA^*∗*^ deceased donor kidney	<0.001	2.12	1.46–3.07	<0.001	3.39	1.83–6.25

Multivariate analysis for graft loss						
Age donor (per year)	0.001	1.02	1.01–1.03	—	—	—
Age recipient (per year)	0.001	0.98	0.97–0.99	0.051	0.98	0.96–1.00
DSA^*∗*^ deceased donor kidney	0.035	2.14	1.06–4.35	0.017	4.94	1.32–18.43

^*∗*^Interaction term for variables.

## Data Availability

The clinical data used to support the findings of this study are included within the article.
